# *Operando* Exploration of CoAl‐LDH: Transformations Driving Alkaline Oxygen Evolution Reaction

**DOI:** 10.1002/smll.202412351

**Published:** 2025-04-02

**Authors:** Mattia Cattelan, Jijin Yang, Leonardo Cielo, Silvia Nappini, Silvia Carlotto, Marco Nalesso, Ilargi Napal Azcona, Rossella Yivlialin, Xiaoming Sun, Gianlorenzo Bussetti, Elena Magnano, Stefano Agnoli

**Affiliations:** ^1^ Department of Chemical Sciences University of Padova via Marzolo 1 Padova 35131 Italy; ^2^ INSTM Istituto Nazionale Scienza e Tecnologia dei Materiali Padova Research Unit Firenze 50121 Italy; ^3^ CIRCC Consorzio Interuniversitario Reattività e Catalisi Padova Research Unit Bari 70126 Italy; ^4^ CNR – Istituto Officina dei Materiali (IOM) S.S. 14 km 163.5 Trieste 34149 Italy; ^5^ Physics Department University of Trieste P.le Europa 1 Trieste 34127 Italy; ^6^ Department of Physics Politecnico di Milano p.za Leonardo da Vinci 32 Milan I‐20133 Italy; ^7^ State Key Laboratory of Chemical Resource Engineering Beijing Advanced Innovation Center for Soft Matter Science Engineering College of Chemistry Beijing University of Chemical Technology Beijing 100029 P. R. China; ^8^ Nanotechnology Research Laboratory Faculty of Engineering University of Sydney Camperdown 2006 Australia

**Keywords:** layered double hydroxide, operando AFM, operando characterizations, operando NEXAFS, oxygen evolution reaction

## Abstract

This work reports a comprehensive study on the morphology, composition, and electronic structure of CoAl layered double hydroxide (CoAl‐LDH) during the oxygen evolution reaction (OER). To capture electrochemically induced transformations, *operando spectroscopic* and *microscopic* methods are combined. The complementary data provided by *operando* near‐edge X‐ray absorption fine structure (NEXAFS), supported by density functional theory (DFT) calculations, and electrochemical atomic force microscopy (AFM), reveal that under OER conditions, CoAl‐LDH is fragmented into smaller particles due to Al leaching. This process forms a “resting” phase with an average Co oxidation state of 2.5+, which readily transforms into the OER‐active β‐CoOOH phase upon further potential increase. This work exemplifies how *operando* methods enable precise tracking of oxidation state changes, element dissolution, and structural transformations at the nanoscale while the electrocatalyst is active. This approach contrasts with conventional pre‐ and post‐mortem characterization, which would instead suggest Co_3_O_4_ formation. These findings extend beyond the specific example of CoAl‐LDH, emphasizing the crucial importance of selective cation leaching, recrystallization, and morphological restructuring, since these processes play a key role not only in designing advanced multi‐element materials but also in understanding the complex nanoscale mechanisms that govern the activation and durability of practical electrocatalysts.

## Graphical abstract

*Operando* spectroscopy and microscopy reveal the dynamic transformations of CoAl layered double hydroxide (CoAl‐LDH) during the oxygen evolution reaction. Al leaching induces fragmentation, forming a “resting” phase with Co^2.5+^, which transitions into β‐CoOOH at higher potentials. These findings underscore the role of selective cation leaching and restructuring in electrocatalysts, providing insights into multi‐element material design.

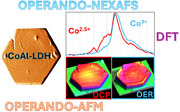

## 1 Introduction

CoAl layered double hydroxide (LDH) is widely utilized in several research fields: in supercapacitors for energy storage given its excellent electrochemical performance,^[^ ^1^ ^]^ in environmental remediation for the adsorption and removal of heavy metals and dyes from wastewater,^[^ ^2^, ^3^ ^]^ in photocatalysis due to a band gap in the visible range and good electron transfer properties,^[^ ^4^ ^]^ and as a precursor for the synthesis of other advanced materials.^[^ ^5^ ^]^ These properties make CoAl‐LDH an exceptionally versatile material suitable for a wide range of applications in the energy and environmental sectors.^[^ ^2^, ^3^, ^4^, ^6^, ^7^, ^8^ ^]^ One of its most studied applications is as an emerging electrocatalyst in electrocatalytic water splitting, particularly facilitating the oxygen evolution reaction (OER).^[^ ^6^, ^7^ ^]^ Therefore, in this work, through advanced spectroscopic and microscopic *operando* techniques, we unveil critical nanoscale insights into the profound transformations CoAl‐LDH undergoes when it is used as an OER electrocatalyst in alkaline conditions.

CoAl‐LDH is composed of positively charged layers and interlayer anions, where Co and Al ions are octahedrally coordinated. Specifically, Co ions have a 2+ oxidation state and are coordinated by six hydroxide (OH^−^) ions, creating Co(OH)_6_ octahedra. Similarly, Al ions, in the 3+ oxidation state, are also surrounded by six hydroxide ions, forming Al(OH)_6_ octahedra. These octahedra share edges within the layers, resulting in a 2D sheet‐like structure, very similar to brucite. CoAl‐LDH shares notable structural similarities with Co(OH)_2_, where Co has also a 2+ oxidation state and forms Co(OH)_6_ octahedra, which likewise connect through shared edges to create layered sheets.^[^ ^9^ ^]^ However, the addition of Al in CoAl‐LDH introduces charge‐balancing mechanisms and interlayer species absent in Co(OH)_2_. The positive charge of these layers is balanced by interlayer anions, such as carbonate or nitrate, along with water molecules.^[^ ^10^ ^]^

A key aspect of this investigation is the study of Al leaching. The incorporated Al ions in the LDH are not stable in alkaline OER conditions^[^ ^11^ ^]^ resulting in significant structural changes during working conditions, on the other hand Co is one of the most studied transition metals given its excellent catalytic activity in OER and high stability in alkaline conditions.^[^ ^12^, ^13^, ^14^ ^]^ More generally, understanding Al leaching is crucial for assessing the structural integrity and performance of other nanoporous materials, such as High Entropy Alloys (HEA). Al leaching can significantly impact on surface area, porosity, and catalytic properties of these materials, influencing their long‐term stability and activity in electrocatalytic applications.^[^ ^15^, ^16^, ^17^ ^]^ Focusing on a binary prototype alloy such as CoAl‐LDH, with its well‐defined structure, allows for a clear observation of the effects of leaching in a simplified system.

In this work, to surpass the practical limits imposed by standard characterization techniques and grasp the true nature of dynamic transformations in electrochemical environment, we investigated CoAl‐LDH using *operando* OER methods. We exploited two crucial *operando* techniques, near‐edge X‐ray absorption fine structure (NEXAFS) and atomic force microscopy (AFM), to get access to complementary information about the chemical state and morphological changes of CoAl‐LDH during OER. To analyze in detail *operando* NEXAFS spectra, density functional theory (DFT) calculations were also performed. Indeed, NEXAFS applied to the L_2,3_‐edge of transition metals is a very sensitive technique for the determination of the oxidation states, and the metal coordination environment,^[^ ^9^ ^]^ which are fundamental descriptors for understanding the functional properties of these materials.^[^ ^18^ ^]^ Transition metal L_2,3_‐edge features arise from states generated by the transition metal‐based 2p → 3d excitations and can be properly modelled using DFT/ROCIS calculations.^[^ ^18^, ^19^ ^]^ In this regard, one of the authors has recently gained considerable experience in the L_2,3_‐edge simulation of several transition metal complexes of V,^[^ ^20^ ^]^ Mn,^[^ ^21^ ^]^ Fe,^[^ ^22^, ^23^ ^]^ and Co.^[^ ^21^, ^24^, ^25^ ^]^

*Operando* AFM investigations prove that, under OER conditions, the material nanosheets retain an overall hexagonal shape at length scale above hundredths of nanometers, although on a shorter scale a fragmentation into smaller nanoparticles can be clearly observed. *Operando* NEXAFS shows that the material at noncatalytic potential adopts a resting phase with an average oxidation state of Co^2.5+^ and active phase of Co^3+^ during OER.

We compared these findings with conventional ex‐situ post‐OER characterization concerning the morphology, atomic and electronic structure,^[^ ^26^, ^27^ ^]^ of the materials. The combined evidence from transmission electron microscopy (TEM), energy dispersive X‐ray (EDX) spectroscopy and NEXAFS highlights a transition from CoAl‐LDH to micrometric Al‐free spinel rectangular shaped Co_3_O_4_ flakes. We believe that the apparent discrepancies between ex‐situ and *operando* characterization arise from the removal of the electrolyte and applied potential, and the extensive drying under ultra‐high vacuum (UHV) conditions, which normally precedes the conventional analysis.

We emphasize that *operando* investigations provide critical insights into the behavior under working conditions of CoAl‐LDH, not directly available through conventional pre‐ and post‐characterizations. The presented *operando* methods allow for precise tracking of oxidation state changes, element dissolution, and structural transformations at the nanoscale while the electrocatalyst is operational, and the same strategy can be applied to other similar materials that dynamically transform during electrochemical processes.

## 2 Results and Discussion

### 2.1 Electrochemical Characterization

**Figure 1:**
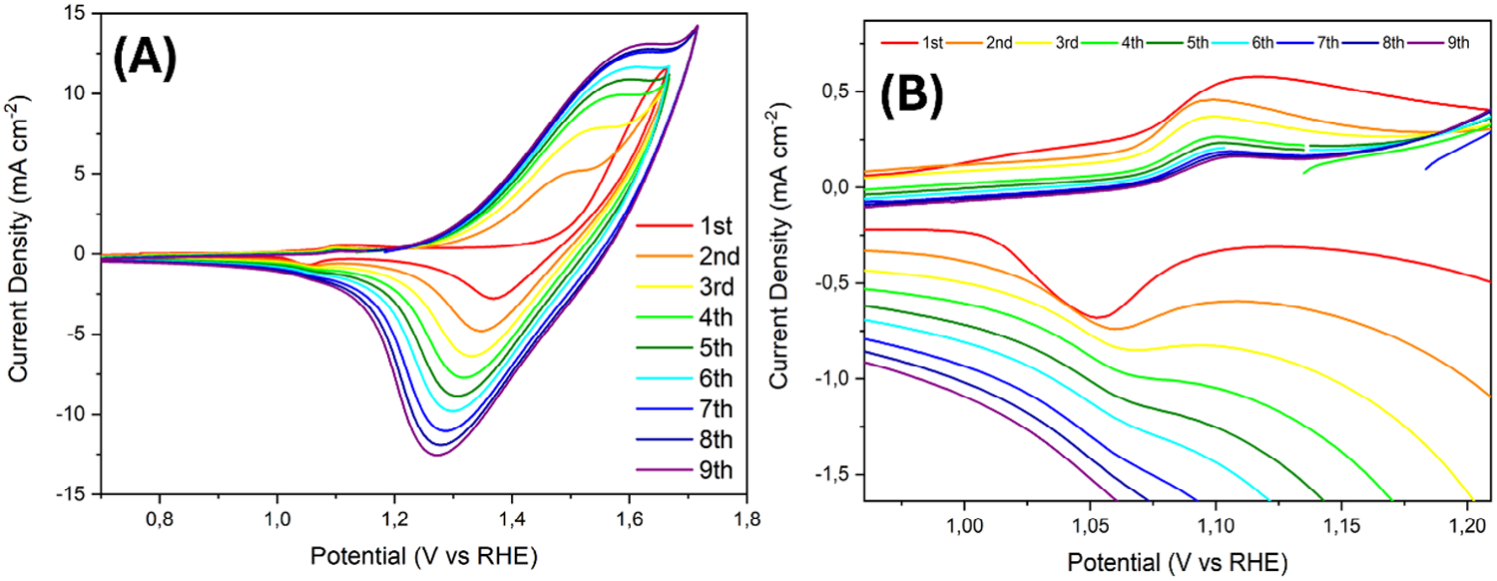
A) Electrochemical characterization of CoAl‐LDH first 9 CVs from 0.7 to 1.7 V versus RHE, at 50 mV s^−1^. B) Inset on the first redox peak.

**Figure** **1**A shows the first nine cyclic voltammograms (CV) of CoAl‐LDH acquired at 50 mV s^−1^ from 0.7 to 1.7 V versus RHE, where two distinct redox pairs are observed.

The redox behavior of β‐Co(OH)₂ has been extensively discussed in the literature^[^ ^28^, ^29^, ^30^, ^31^, ^32^ ^]^ with Mefford et al. recently providing an explanation based on *operando* studies,^[^ ^9^ ^]^ which has been used here as a basis for comparison. For β‐Co(OH)_2_, the first redox peak at 1.1/1.05 V versus RHE (Figure 1B) involves the formation of a water intercalated phase. This process has been already reported for other hydroxide systems such as α‐Ni(OH)_2_^[^ ^33^ ^]^ and can be described according to the reaction:

(1) $$\beta -\mathrm{Co}{\left(\mathrm{OH}\right)}_{2}+0.5\mathrm{OH}\to \alpha -\mathrm{Co}O_{2}H_{1.5}\cdot 0.5H_{2}O+0.5e^{-}$$

However, unlike β‐Co(OH)₂, Al in CoAl‐LDH can easily leach when exposed to an oxidizing alkaline environment. This explains the irreversible features observed in the CV, where the first redox peak at ≈1.1 V progressively decreases with CV cycling. We infer that CoAl‐LDH is rapidly affected by Al leaching, with metal dissolution taking place during the very first CV cycle, as subsequently corroborated by *operando* NEXAFS and AFM techniques. This is evidenced by the rapid shift in the centroid of the peak during the first CV, compared to the successive cycles. This phenomenon may also facilitate the stabilization of water intercalation and the formation of α‐CoO₂H₁.₅ · 0.5H₂O within the same potential window. This water‐intercalated phase with an overall oxidation state of +2.5^[^ ^9^ ^]^ was observed as stable resting phase by *operando* NEXAFS (see paragraph 2.3.1) and contributes to the irreversible lifting of the flake edges (see paragraph 2.3.2).

For both CoAl‐LDH and β‐Co(OH)_2_, the second redox couple, at ≈1.4/1.5 V is linked to the transformation that produces the active OER species, i.e., the cobalt oxyhydroxide, following this reaction:

(2) $$\alpha -\mathrm{Co}O_{2}H_{1.5}\cdot 0.5H_{2}O\to \beta -\mathrm{CoOOH}+0.5H_{3}O^{+}+0.5e$$

The signal related to this second redox couple increases rapidly with CV cycling, in contrast to the first pair. This indicates a probable activation process of the basal plane of the material, which we visualize using *operando* AFM, as discussed below in Section 2.3.2.

Thus, we can approximate that for CoAl‐LDH, irreversible Al dissolution likely occurs ≈1.1–1.3 V, coinciding with the formation of a water intercalated phase (α‐CoO_2_H_1.5_· 0.5H_2_O). The resulting defects stabilize a structure that supports a single transition, from an average oxidation state of Co^2^ ^.5+^ to Co^3^⁺. This transition significantly increases with CV cycling, thereby enhancing the material catalytic activity in the OER, explaining the increased activity compared to β‐Co(OH)_2_ (Figure S2A, Supporting Information). In order to visualize the difference in OER activity of CoAl‐LDH before and after Al leaching, we performed linear sweep voltammetry (LSV) at lower pH, i.e., in 0.05 m KOH, to slow down the massive leaching that takes place at high electrochemical potential. Indeed, in 0.1 m KOH, as discussed in the next paragraphs, the leaching is so fast that most of Al dissolution occurs as soon as catalytic conditions are reached for the first time, obtaining LSVs with no noticeable differences before and after electrochemical (EC) aging (Figure S2B,C, Supporting Information). In 0.05 m KOH, the leaching is still relevant but slower and allows observing a progressive improvement in the OER activity during accelerated EC aging, as the Al dissolution proceeds. To roughly estimate the change in surface area, CV scans were recorded in a non‐Faradaic region close to the open circuit potential (OCP) (Figure S2D, Supporting Information) and the results indicate a substantial increase in capacitance after EC aging. This increase is related to the formation of new catalytically active sites, such facets, edges and undercoordinated cations produced by Al leaching, as it will be discussed in the *operando* AFM section (see paragraph 2.3.2).

### 2.2 As‐Prepared Material Characterization

**Figure 2:**
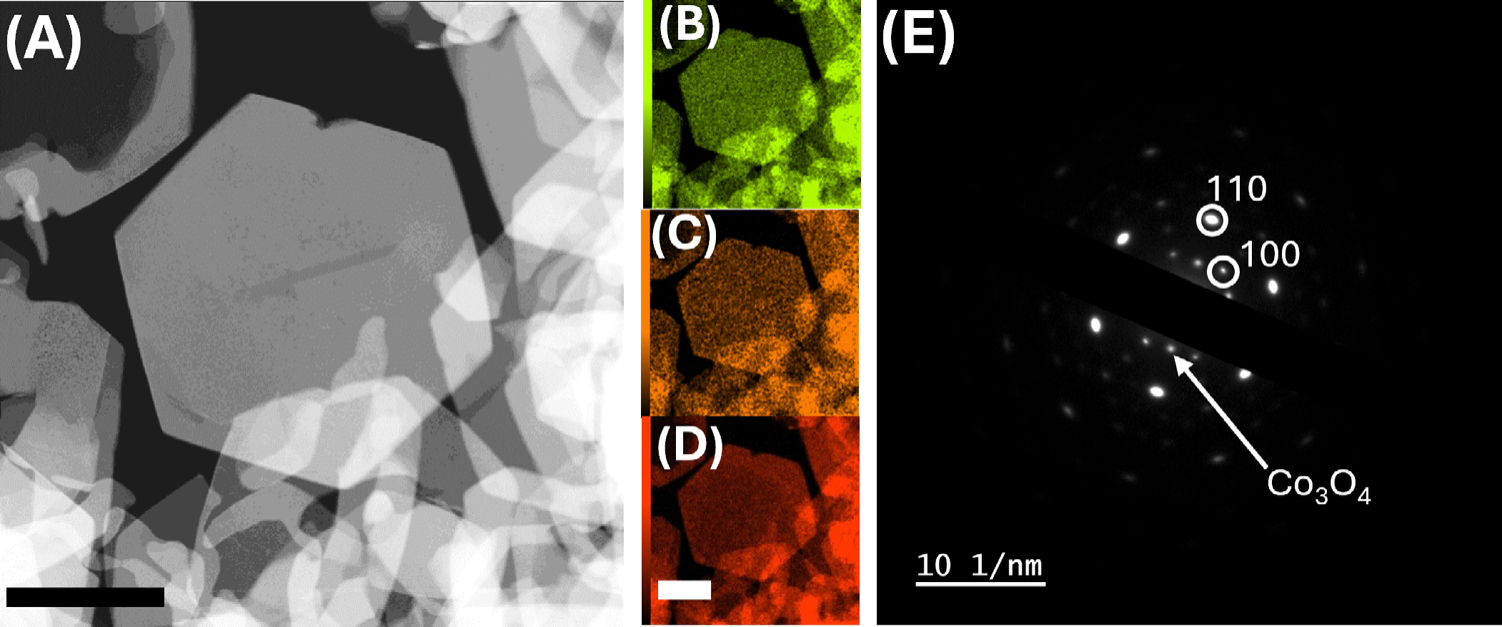
TEM panel. A) Secondary electron image of CoAl‐LDH as‐prepared, B) Co K edge map, C) Al K edge map, D) O K edge map, all scale bars: 1 µm E) SAED with indexed spots and indicated the reflex of Co_3_O_4_ due to beam‐induced effect is much less bright than the main spots of CoAl‐LDH (indicated as (110)).

TEM imaging and EDX spectroscopy were used to investigate the morphological and structural properties of the pristine CoAl‐LDH. To obtain genuine information, a suspension of the pure, pristine materials was directly drop casted on a TEM grid without the addition of conductive carbon or Nafion. In **Figure** **2**A, the CoAl‐LDH nanosheets are well‐recognizable in the form of highly crystalline and regular hexagonal flakes, ≈1 µm wide and various fragments with characteristic edges crossing at an angle of 120°. Smaller flakes with rounded edges are also visible, likely resulting from the specific synthesis process, which involves metal etching during growth due to in‐situ urea hydrolysis.^[^ ^34^ ^]^ Figure 2B–D reports O K_α_, Al K_α_ and Co K_α_ EDX chemical maps. The measured Co: Al signal ratio is 3:1, consistent with the stoichiometric ratio between the metal precursors used in the synthesis. Structural imperfections such as edge cracks and grain boundaries do not affect the Co/Al distribution. Selected area electron diffraction (SAED), in Figure 2E, confirms the expected CoAl‐LDH structure.^[^ ^35^ ^]^ Additional spots in the diffraction pattern indicate a partial minoritarian transition to Co_3_O_4_ induced by electron beam irradiation during TEM.^[^ ^36^ ^]^

**Figure 3:**
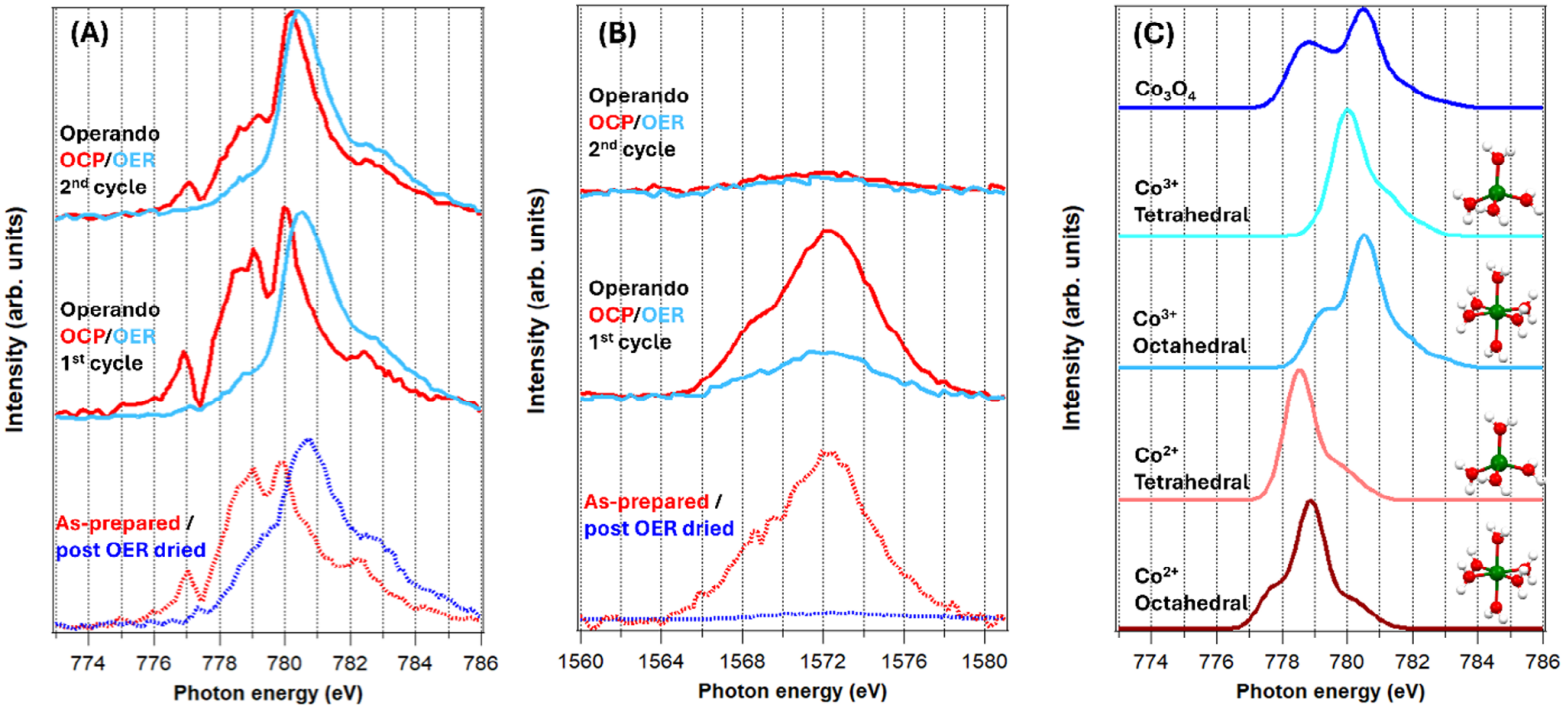
*Operando* and ab‐initio NEXAFS. A) From the bottom: Co L_3_ edge fluorescence spectra of the as‐prepared sample (red) and the ex‐situ EC aged and dried sample (blue), first *operando* EC cycle OCP (red) and OER (light blue), and second EC cycle OCP (red) and OER (light blue). B) From the bottom: Al K edge fluorescence spectra of the as‐prepared sample (red) and the EC aged and dried sample (blue), first *operando* EC cycle OCP (red) and OER (light blue), and second EC cycle OCP (red) and OER (light blue). C) Series of simulated spectra from the bottom, Co^2+^ octahedrally coordinated by oxygen (dark red), Co^2+^ tetrahedrally coordinated by oxygen (pink), Co^3+^ octahedrally coordinated by oxygen (light blue), Co^3+^ tetrahedrally coordinated by oxygen (cyan) and Co_3_O_4_ (blue). A Gaussian broadening factor of 1.0 eV has been applied to all simulations.

NEXAFS spectra at Co L_2,3_‐, O K‐, and Al K‐edges for the pristine materials, measured in Flourescence Yield (FY) mode are reported in **Figure** **3**A,B (red dashed lines) and Figure S3 (Supporting Information) (conventional NEXAFS). By Co L‐edge NEXAFS spectra, the oxidation states, and the coordinative environment (tetrahedral or octahedral) of Co atoms are deduced, as they specifically probe the electron distribution within the redox‐active Co 3d states (Co 2p →3d transitions). The Co L_2,3_ absorption spectrum clearly shows the presence of Co^2+^ ions as suggested by two bright lines at 779 and 780 eV and relative intensity of the small shoulders at either side. This aligns well with the structure of CoAl‐LDH, which comprises octahedral Co^2+^ cations with a structure similar to β‐Co(OH)_2_.^[^ ^9^ ^]^

The Al K‐edge shows the typical features of the gibbsite structure, aligning with the CoAl‐LDH structure with Al in octahedral AlO_6_ units,^[^ ^37^, ^38^ ^]^ whereas, the O K‐edge is similar to that measured on β‐Co(OH)_2_ and reported in Figure S4B (Supporting Information), with the addition of a pre‐peak at ≈530 eV, likely associated with Al‐OH, also observable in the gibbsite structure.^[^ ^37^, ^38^ ^]^ Overall the NEXAFS data confirm the presence of CoAl‐LDH.

### 2.3 Operando Characterization

#### 2.3.1 Ab‐Initio Calculations and *Operando* NEXAFS

To gain information on the changes in oxidation states and the chemical environment of Co cations, which were identified in the electrochemical characterization carried out by CV, the Co L_3_ measured spectra for the dried samples (as‐prepared and ex‐situ after OER) and under *operando* conditions (Figure 3A) were compared to other reference compounds and *operando* findings (see Figures S4 and S5, Supporting Information) and to the simulated spectra for Co^3+^ and Co^2+^ in tetrahedral and octahedral coordination, as well as to Co_3_O_4_ spinel provided by ab‐initio calculations (see Figure 3C).

Simulated spectra of Co^2^⁺ and Co^3^⁺ octahedrally coordinated with an oxygen atom directly bonded to the metal (Figure 3C) show a quite similar shape with different energy positions of the main peak, i.e., 779.0 and 780.5 eV for Co^2+^ and Co^3+^, respectively. Both show two distinct lower‐intensity features at ≈1.5 eV above and below the main line, (see also the calculations with a smaller Gaussian broadening for octahedrally coordinated Co^2+[^ ^39^ ^]^ in Figure S6, Supporting Information). Similarly, the simulated spectra for tetrahedrally coordinated Co^2^⁺ and Co^3^⁺ exhibit comparable shapes, but are shifted in energy, with main peaks at 778.5 and 780.0 eV for Co^2^⁺ and Co^3^⁺ respectively. They also display a single, lower‐intensity feature ≈1 eV above the main peak.

Based on purely experimental data, the comparison of our results with the reference spectra in Figure S4 (Supporting Information), and particularly with the *operando* spectra measured on β‐Co(OH)_2_,^[^ ^9^ ^]^ (Figure S5, Supporting Information) suggests that our *operando* measurements in Figure 3A can be interpreted by assuming a mixture of Co^2^⁺ and Co^3^⁺, both octahedrally coordinated, as also reported in ref. [9] for β‐Co(OH)_2_. This interpretation, therefore, assumes the absence of more oxidized species such as Co^4+^, which would exhibit a fingerprint at ≈1 eV higher photon energy than Co^3+^.^[^ ^9^ ^]^ Indeed, the strong similarity between our CoAl‐LDH and the reference β‐Co(OH)_2_ spectra^[^ ^9^ ^]^ (Figures S4 and S5, Supporting Information) makes it highly unlikely the presence of any significant oxidation in the starting materials.

To extract the average oxidation state of the material, the components A and B, representing the fractions of Co^2^⁺ and Co^3^⁺ respectively (with values ranging from 0 to 1), are summed together, varying the fraction of the components from 0 to 1 in steps of 0.01. The best combination is found by minimizing the square difference with the experimentally observed spectra. The average oxidation state can be calculated as follows:

(3) $$\mathrm{Average}\;\mathrm{oxidation}\;\mathrm{state}=\left(A*2+B*3\right)/\left(A+B\right)$$

We chose two independent sets of components for this analysis: 1) Our own data, specifically the spectra of as‐prepared CoAl‐LDH for Co^2^⁺ (component A) and the *operando* OER spectra in Figure 3A for Co^3^⁺ (component B), and 2) the reference spectra from the *operando* measurements in ref. [9], where Figure S5A (Supporting Information) representing Co^2^⁺ (component A) and Figure S5C (Supporting Information) representing Co^3^⁺ (component B). By employing these two independent data sets, we calculated the average oxidation states with an error margin of ±0.1. An example of this calculation for the OCP measurement during the 1^st^ cycle (Figure 3A) is provided in Figure S7 (Supporting Information).

As shown by EC measurements in Figure 1, it is crucial to understand the changes in the material during the very first exposure to oxidizing alkaline conditions. Therefore, the Co L_3_‐edge spectra for the first and second CV cycles are reported in Figure 3A. Focusing on the first cycle, it is important to note that for *operando* NEXAFS, the sample was held at the OCP (0.90 V) in contact with the electrolyte for a few hours before starting the measurements. Under these conditions, the average oxidation state for the sample at the OCP was found to increase slightly to 2.1+ (see Figure S7, Supporting Information). A noninteger oxidation state number indicates a nonnegligible presence of Co^3^⁺, visible by the different ratio of the intensity of the signals at 779 and 780 eV compared to the as‐prepared sample, which we assume to be pure Co^2^⁺, see Figure 3A and Figure S4 (Supporting Information). From previous literature works at OCP conditions, Co should be in the 2+ oxidation state.^[^ ^9^ ^]^ The observed slight oxidation of some cations to Co^3+^ during the first cycle was probably justified by a small dissolution of Al due to the prolonged exposure time of the sample to KOH,^[^ ^40^ ^]^ even though the molarity of 0.1 m was chosen to limit Al dissolution. However, it must be pointed out that the Al leaching is minimal because the Al K‐edge (Figure 3B) is still clearly detectable with an adsorption edge consistent with the gibbsite structure, as seen for the as‐prepared sample (Figure S3B, Supporting Information). An alternative hypothesis is that the surface of the sample is transitioning into a more complex mixed system with Co^3^⁺ and Co^2^⁺ ions.^[^ ^41^ ^]^ This explanation will be discussed in more detail later, specifically in relation to the second cycle experiment performed during *operando* measurements.

The comparison with simulated spectra is particularly useful for understanding the differences observed between conventional ex‐situ characterization and *operando* measurements of the active OER species. The *operando* measurements were obtained by polarizing the sample at 1.7 V and allowing it to stabilize for 30 min. The two main candidates for OER active species are the β‐CoOOH with a Co^3+^ octahedrally coordinated and the spinel phase (Co_3_O_4_), which several authors report to be stable in OER conditions^[^ ^26^, ^27^ ^]^ and indeed can be identified by the post‐mortem characterization of our own materials using TEM images and NEXAFS, see ex‐situ post‐mortem characterization paragraph (see paragraph 2.4). The experimental OER spectra in the first and second OER cycle on one hand are similar to that of the simulated octahedral Co^3+^ ions in terms of energy position and relative intensity of the different features, on the other hand they are less consistent with a Co_3_O_4_ fingerprint, featuring the simultaneous presence of Co^3^⁺ octahedrally and Co^2^⁺ tetrahedrally coordinated species in a 2:1 ratio.^[^ ^42^, ^43^ ^]^ (see Figure S8, Supporting Information). Therefore, also the simulated spectra in Figure 3C point that our active species is mainly Co^3^⁺ octahedrally coordinated and coherent with previous *operando* work on β‐Co(OH)_2_ sample (Figure S5C, Supporting Information)^[^ ^9^ ^]^ validating the hypothesis used to calculate the average oxidation state above.

Regarding the Al‐K edge, Figure 3B shows that the signals are strongly decreased at the very first OER, reaching ≈30% of the starting point and decreasing further with the second CV until stabilizing at 6%, as also reported in ex‐situ measurements, Figure S3B (Supporting Information) and Figure 3B. We associate the rapid leaching of Al in our conditions with the irreversible change observed in the EC in Figure 1.

The second cycle is representative of the final phases of the material, as the measurement time was long enough to stabilize the sample structural changes. We expect that the catalyst's structure is strongly affected by Al leaching, consistently with previous reports.^[^ ^11^ ^]^ Indeed, using the calculations described above, at the second OCP we found a Co phase with an average oxidation state of 2.5+, remaining stable throughout subsequent cycles when the sample was set at OCP or reducing potentials (0.6 V). An average noninteger oxidation state of 2.5+ supports the co‐presence of Co atoms with Co^3+^ and Co^2+^ oxidation states in an equal amount. This average oxidation state of 2.5+ precisely matches previously reported data on β‐Co(OH)_2_ and has been associated with the α‐CoO_2_H_1.5_ · 0.5H_2_O system,^[^ ^9^ ^]^ see (Figure S5B, Supporting Information). This new “resting” phase, characterized by a distinctive average oxidation state, explains the significantly damped redox peak for CoAl‐LDH observed in the CV cycles (Figure 1). In β‐Co(OH)_2_, this phase was identified as an intermediate state between Co^2+^ (i.e., β‐Co(OH)₂) and Co^3+^ (i.e., β‐CoOOH),^[^ ^9^ ^]^ that is made possible by the OH^−^ intercalation in β‐Co(OH)_2_, forming the intercalated water phase α‐CoO_2_H_1.5_·0.5H_2_O. At higher potentials, this intermediate structure can be converted to the active phase β‐CoOOH, through the elimination of intercalated water layer and concurrent Co^2+^ to Co^3+^ transition. Consequently, β‐Co(OH)₂ must undergo two oxidation state transitions to be converted into the catalytically active β‐CoOOH phase. However, in our case, we propose that the highly defective structure resulting from Al leaching stabilizes the intercalated phase even at the OCP, allowing the sample to transition directly to the active state (Co^3+^) at a potential of ≈1.7 V.^[^ ^9^ ^]^ This unusual resting phase, with a 2.5+ oxidation state, can also be linked to the defective CoO_x_(OH)_y_ phase presented by Bergmann et al.^[^ ^41^ ^]^ where it is reported that the crystal structure of Co^2+^ oxides exhibited limited integrity at the surface during OER, as they tend to transform toward 3D cross‐linked CoO_x_(OH)_y_ with twofold metal‐coordinated oxygen atoms bridging pairs of fivefold oxygen‐coordinated high spin Co^3+^ and Co^2+^ ions.^[^ ^33^ ^]^

The OER active phase for the second cycle is identical to the first, confirming that Co^3^⁺ (β‐CoOOH) serves as the active site in the OER,^[^ ^9^, ^41^, ^44^, ^45^ ^]^ even after a complete Al leaching. Differences between the active phase identified in our work and that found in previous literature^[^ ^41^ ^]^ may be attributed to the alkaline environment used in this study. Despite the approximations used in the NEXAFS analysis to calculate the average oxidation states of cations, the consistent absence of intense signals at higher photon energies allows us to exclude the presence of Co^4+^.^[^ ^32^, ^46^ ^]^ This remains true despite the substantial number of defects and the morphological changes in the sample, which have been carefully investigated by *operando* AFM.

#### 2.3.2 *Operando* AFM

The morphological changes of the material were studied by *operando* AFM, which allows precise monitoring of the sample morphology. For these measurements, it was decided to proceed using a potentiostatic approach, i.e., the potential applied to the sample is modified between scans, while it remains constant during the image acquisition, which lasted 512 seconds.

**Figure 4:**
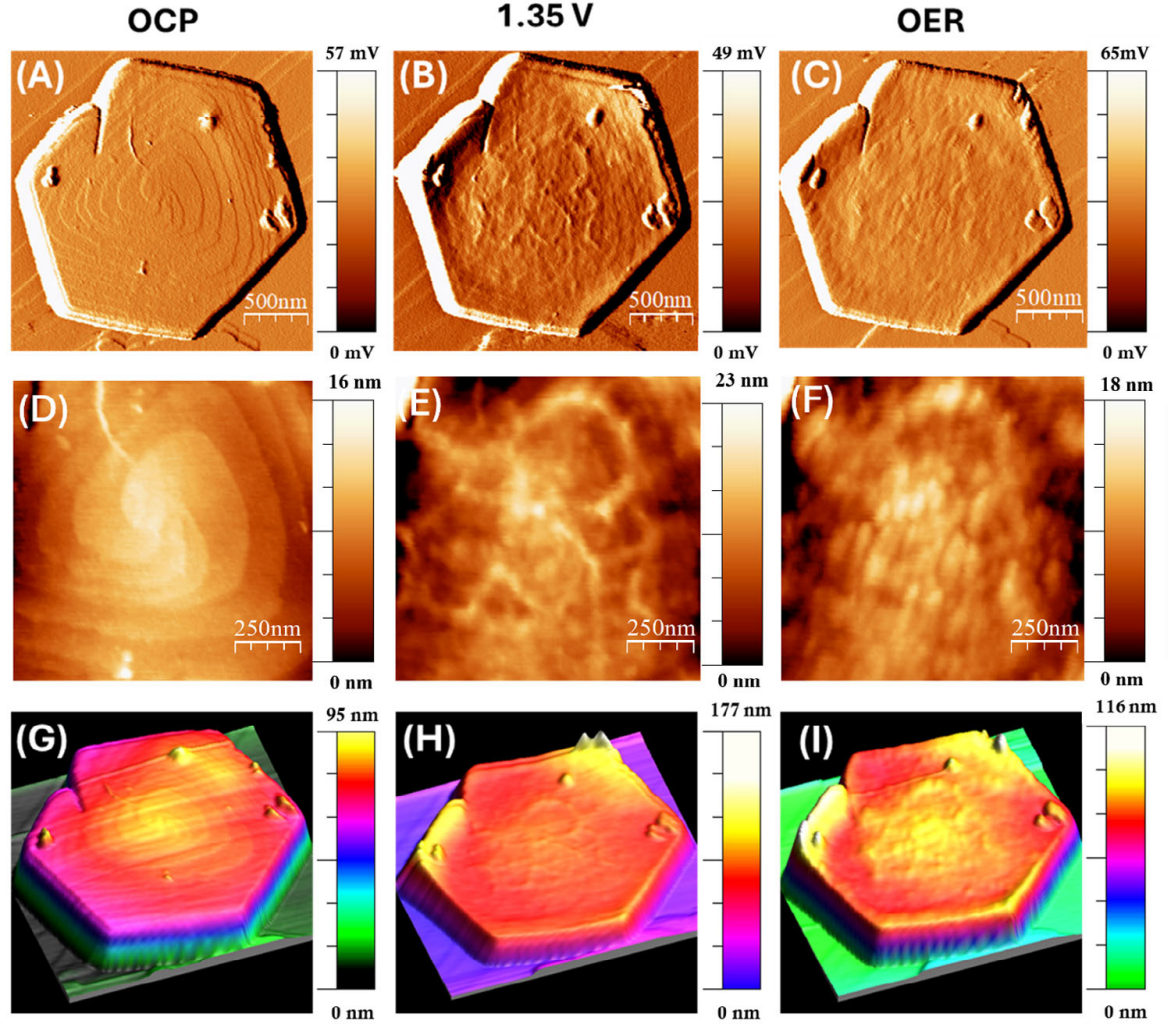
*Operando* AFM images of a representative flake of CoAl‐LDH deposited on the HOPG surface. A,D,G) Images acquired at OCP (0.8 V). B,E,H) Images acquired at 1.35 V. C,F,I) Images acquired at OER (1.75 V). Panels (A,B,C) report the amplitude signal; panels (D,E,F) show a zoom in of the topography at the center of the flake; panels (G,H,I) show the 3D topography in false colors.

The AFM images collected at the OCP (0.8 V) (**Figure** **4**A,D,G) give a clear picture of the presence of hexagonal flakes with a side dimension of ≈1 µm. The synthesis of CoAl‐LDH under hydrothermal conditions without stirring^[^ ^47^ ^]^ produces a growth mode mediated by screw dislocations. This characteristic line defect is visible at the center of the flake (Figure 4D), as evident from a series of concentric, raised steps that spiral outward from a central point. The step heights extracted from the topographic images are less than 1 nm, consistent with the height of a single LDH layer,^[^ ^47^ ^]^ see Figure S9A,B (Supporting Information). The presence of this type of dislocation suggests that the observed flake is most probably a single crystallite of LDH,^[^ ^47^, ^48^ ^]^ and not a vertical stack of flakes, which could hinder the electrical contact from the highly ordered pyrolytic graphite (HOPG) substrate during EC measurements.^[^ ^49^ ^]^

The screw dislocation is stable in the potential range from the OCP to1.3 V, whereas just above this potential (Figure 4B,E,H), we observe the formation of new structures at the center of the flake, which modify the surface morphology. These newly created features are localized around the dislocation and extend outward for few nanometers (Figure S9, Supporting Information). Even though the atomic resolution is not possible, AFM topographies indicate the formation of nanometric globular features with an apparent corrugation of 1–2 nm, which sometimes connect to each other forming linear structure, aligned with pre‐existing spiraling edges of the flake (Figure 4B,E; Figure S9, Supporting Information), Conversely, on the outward periphery of the flake, the spiral stepped structure of the dislocation is still clearly recognizable.

As discussed above in the description of the electrochemical properties, we expect an irreversible redox process ≈1.3 V, i.e., before the second redox peak that leads to the OER active phase, which is accompanied by some Al leaching. Therefore, we link the formation of these rough areas to a significant structural rearrangement of the LDH consisting in the fracturing and redeposition of material of the existing flat and single crystalline flake surface, which is a consequence of the dissolution of Al and the formation of Al‐depleted small CoO_x_H_y_ particles. The dissolution of Al as AlO^2−^ starting from the center of CoAl‐LDH nanosheets in alkaline conditions is reported to be the early stage of the formation of LDH with nano‐ring morphology.^[^ ^34^ ^]^ Although the overall hexagonal shape is macroscopically maintained, at the nanoscale numerous newly formed structures are observed by AFM at the center of the flake, indicating fragmentation into smaller particles.

**Figure 5:**
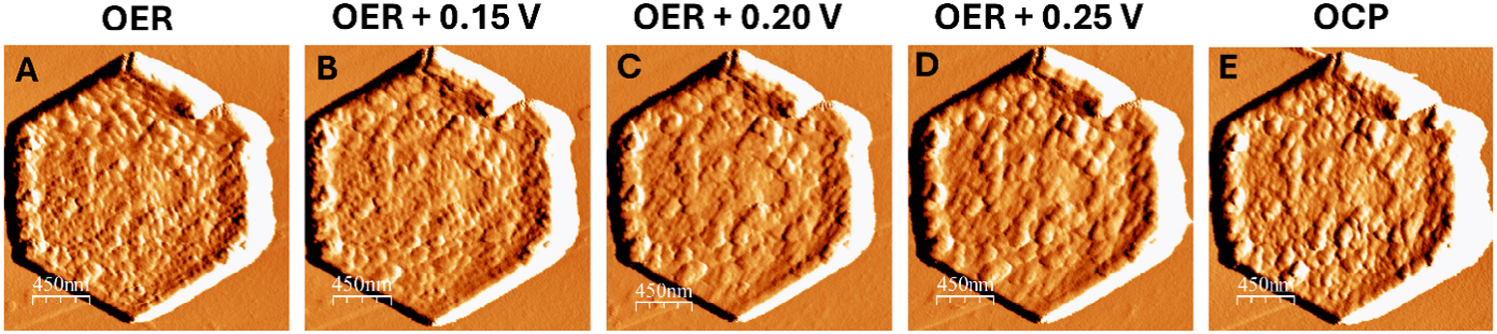
*Operando* AFM panel. Images in amplitude A) at OER (1.75 V), B) images at 1.9 V, C) at 1.95 V, D) at 2.00 V, E) at OCP after prolonged OER. Panel (A,B,C) images in amplitude, (D,E,F) topography of the center of the flake.

Given that the onset potential for the structural modification of the LDH, i.e., 1.35 V, roughly coincides with the potential for water intercalation and formation of α‐CoO_2_H_1.5_ · 0.5H_2_O^[^ ^9^ ^]^ it is not easy to separate the two phenomena. However, we can infer a vertical expansion of the material because, at 1.35 V and above, a sharp increase in the height of edges is clearly visible from Figure 4H and line profiles in Figure S9 (Supporting Information). This behavior is similar to what was reported in another study focusing on the closely related β‐Co(OH)_2_.^[^ ^9^ ^]^ Thus, we could attribute the edge rise to water intercalation. Interestingly, in our case, this transformation is irreversible and remains evident at OCP after OER (see particle's edge in **Figure** **5**; Figure S10, Supporting Information). We propose that this irreversibility of water intercalation is linked to defective α‐CoO_2_H_1.5_ · 0.5H_2_O. This phase has an average oxidation state of 2.5+, and this specific oxidation state was observed as the stable resting phase by *operando* NEXAFS (Figure 3A).

In OER potential window, above 1.65 V (Figure 4C,F,I), the surface morphology becomes increasingly corrugated, and the screw dislocation is no longer observable, not even at the borders. This overall change in morphology indicates the probable complete dissolution of Al and the full transformation into the active OER phase, specifically β‐CoOOH. The linear structures formed at 1.35 V are still visible at higher EC potentials during OER (see Figure 4E,F), and they appear to facilitate the nucleation of globular particles that grow from a diameter of ≈40 nm at 1.35 V to ≈60 nm at 1.65 V.

Under OER potential, another hexagonal flake was found, see Figure 5. The morphology of this flake was already deeply changed with respect to an as‐prepared sample because it had the same EC aging as the flake analyzed in Figure 4, indeed it showed an overall rough morphology. The flake remained stable up to the highest tested potential, i.e., 0.25 V above OER namely 2V. Following this test, the potential was decreased until reaching OCP conditions.

Other studies report that in strongly catalytic conditions, the AFM topography becomes rough and shows new globular features due to the nucleation of gas bubbles. However, we can safely associate the topographical changes observed during OER with a genuine structural transformation of the sample rather than the creation of oxygen bubbles^[^ ^50^ ^]^ because such features do not have a transient existence and remain stable also when the potential is swept back to the OCP conditions. (see Figure 5D,E; Figure S11, Supporting Information).

For the images in Figure 5, we could calculate the average particle size, but this analysis is particularly difficult due to the heterogeneity of the surface and multimodal distribution of the particles that increase the uncertainty related to the area. The average area of the fragmented particles was calculated to be 3.4 ± 2.2, 4.4 ± 2.9, 4.5 ± 3.2, and 5.3 ± 4.2 ×10^−3^ µm^2^ for Figure 5A,B,C,D, respectively. For Figure 5E, at the OCP, the average area was 3.8 ± 2.7 nm^2^, but the smaller value could be ascribed also to the better lateral resolution of *operando* AFM, at no catalytic potential. Indeed, the poorer lateral resolution during gas evolution (i.e., OER conditions) is likely related to the dynamic evolution of gas, which interferes with the resolution of the image.^[^ ^51^ ^]^ However, we believe that we are not directly visualizing oxygen‐adsorbed bubbles, as explained above. Nonetheless, there is an increase in the particle size from OER condition to 2 V, indicating the growth of these particles. This analysis can be further supported by considering the number of particles per µm square. This value decreases by ≈15% passing from the initial pictures to the final measurements at the OCP. This trend suggests also that smaller particles coalesce to form larger ones. Considering that the main hexagonal flake was ≈2.1 µm^2^, the size of a single particle corresponds approximately to a decrease of 390–620 times, which is much larger than what is reported in the literature.^[^ ^11^ ^]^ However, in the present study, a much more accurate analysis has been conducted, by keeping into account fragments that still form a macroscopic single grain.

After the potentiostatic experiment summarized in Figure 5, we proceeded to test 50 CV cycles from OCP to OER, while imaging *operando* with AFM; both the macroscopic hexagonal shape, as well as the newly formed particles remained constant (see Figure S12, Supporting Information).

It must be noted that it is more difficult to observe the lateral contraction/expansion of the flakes, as observed in other LDHs^[^ ^50^ ^]^ and β‐Co(OH)_2_^[^ ^9^ ^]^ whose reason was traced back to a topotactical transformation that imparts a large strain into a coherent crystalline material. In this case, however, we think that the dissolution of Al allows strain relaxation in the flakes by creating defects and especially grain boundaries. Interestingly, the cracking of the lattice at the center of the flake with nucleation and growth of smaller particles can be the reason for the enhanced OER activity compared to conventional β‐Co(OH)_2_ (Figure S2, Supporting Information).

### 2.4 Ex‐Situ Post‐Mortem Characterization

To emphasize the importance of the methodological approach based on operando techniques, it is quite instructive to compare the previous investigation with the data that can be obtained through the post‐mortem characterization of our samples through standard techniques such as TEM, electron diffraction, XPS and XRD. To simulate extensive EC work, accelerated aging treatments consisting of 50 CVs from 0.7 to 1.7 V at 50 mV s^−1^ were used.

TEM analysis, reported in Figure S13 (Supporting Information), show morphological and structural changes compared to the pristine CoAl‐LDH flakes. Some hexagonal flakes are still visible, but most flakes show a clear transformation into rectangular shapes characterized by sharp edges forming 90° angles. EDX chemical and spectra maps (Figure S13B,C,D,F, Supporting Information) on these flakes, show only Co and O related signals whereas Al features are completely absent, proving that it was leached.^[^ ^11^ ^]^ SAED performed on those flakes shows a distinctive rectangular diffraction pattern that can be associated with the spinel structure of Co_3_O_4_.^[^ ^52^ ^]^ The remaining unchanged hexagonal flakes, on the other hand, exhibit an unaltered ratio of Co:Al 3:1 and CoAl‐LDH diffraction pattern. The inspection of several TEM images suggests that most flakes have transitioned into the new Co_3_O_4_ phase. However, we attribute the persistence of some hexagonal flakes to poor electrical contact between the working electrode, i.e., the TEM grid, and individual flakes which are intrinsically poorly conductive. In conventional EC or space‐averaged acquisitions, this poor conductivity is typically mitigated by the insertion of a conductive medium (e.g., carbon) and polymers. However, we did not follow this approach for this characterization to obtain a clear view of the LDH and to maintain thin films, avoiding bulky aggregates that could affect the TEM images. A similar heterogeneous response to EC aging was reported previously during the *operando* investigation of β‐Co(OH)_2_ electrodes.^[^ ^49^ ^]^

NEXAFS spectra at Co L_2,3_‐, O K‐, and Al K‐edges for the ex‐situ EC‐aged dried samples, measured in FY mode are reported in blue in Figure S3 (Supporting Information). The samples were deposited on gold substrates with conductive carbon and Nafion to favor EC transition on all the flakes, avoiding the heterogeneous phase as seen in TEM. The Co L_23_‐edge undergoes a significant variation, transitioning from a predominantly Co^2+^ state, as expected for the as‐deposited sample with a structure similar to Co(OH)_2_,^[^ ^9^ ^]^ to Co_3_O_4_^[^ ^53^ ^]^ after EC aging, confirming the TEM results. The Co L_2,3_ spectra are comparable with the NEXAFS standard of Co(OH)_2_ (Co^2+^) for the as‐prepared and spinel Co_3_O_4_^[^ ^54^ ^]^ for the EC‐aged, see Figure S4A (Supporting Information).^[^ ^35^, ^36^ ^]^ After OER aging, the Al signal experiences a substantial intensity decrease, amounting to ≈5% of the as‐prepared sample signal, as previously reported for this material^[^ ^11^ ^]^ and consistent with the Al Pourbaix diagram.^[^ ^55^ ^]^

O K‐edge adsorption line also undergoes notable modifications after OER aging,^[^ ^35^, ^36^ ^]^ showing a peak at 530.4 eV photon energy, which can be ascribed to the formation of Co_3_O_4_, see Figure S4B (Supporting Information).^[,^ ^41^, ^52^, ^56^, ^57^ ^]^ However, it is crucial to note that interpreting the oxygen line for our samples is challenging due to the contribution of C‐O bonds from Nafion and conductive carbon.

In conclusion, the ex‐situ post‐mortem characterization would correctly suggest a massive Al leaching, but accompanied by structural transition from CoAl‐LDH to Co_3_O_4_ after EC accelerated aging treatment. This conclusion would be also reinforced by complementing the characterization with other conventional non‐*operando* techniques such as X‐ray photoelectron spectroscopy (XPS) and X‐ray diffraction (XRD), as shown in Figure S14 (Supporting Information), which both confirm the identity of Co_3_O_4_ in the aged catalyst. Interestingly, an amorphous structure with the presence of very faint XRD reflections due to β‐Co(OH)_2_ and β‐CoOOH is observed when drying is incomplete,^[^ ^11^ ^]^ confirming that the sample had not fully dried from the electrolyte and was likely only beginning to transition to the more stable spinel phase.

## 3 Conclusion

In our work, CoAl‐LDH has been investigated as an alkaline OER electrocatalyst. We demonstrate through *operando* NEXAFS experiments, that the active phase of this material during the OER is β‐CoOOH, while at the OCP it adopts a resting phase with an average oxidation state of 2.5+. Using *operando* AFM, we visualized that the sample retains a macroscopic hexagonal shape, but shows a great fragmentation inside the basal plane, which starts at potential values lower than those required for the OER. These measurements provide a rather different picture from the conventional post‐mortem characterization, which conversely points to the formation of the spinel cubic phase of Co_3_O_4_ after OER treatment. However, the spinel phase was the result of extensive drying of the defective Al‐depleted CoO_x_H_y_, which naturally recrystallized into the most stable oxide,^[^ ^52^, ^58^ ^]^ as also confirmed by XRD.

By combining *operando* techniques, we gain critical insights into the material properties that are not directly accessible through conventional pre‐post ex‐situ characterization. The well‐known enhanced OER activity compared to the parent material, β‐Co(OH)_2_, can be explained by the fragmentation triggered by the Al leaching. This morphological transformation combined with the presence of an oxidative electrochemical environment leads to the stabilization of a mixed oxidation state defective phase that facilitates the transition to β‐CoOOH during the OER and increases the number of exposed active sites.

We think that our *operando* characterization methodology on CoAl‐LDH and the results highlighted in this work can also be applied to different electrocatalysts that exhibit intrinsic leaching of elements and creation of nanostructured defects, such as several HEA^[^ ^15^, ^17^ ^]^ and multi metal oxides.^[^ ^59^ ^]^

## 4 Experimental Section

### Material Preparation

CoAl‐LDHs were synthesized by a hydrothermal method. First, Co(NO_3_)_2_·6H_2_O (0.75 mmol), Al(NO_3_)_3_·9H_2_O (0.25 mmol), and CO(NH_2_)_2_ (20 mmol) were dissolved in 40 mL of deionized water (chemicals from Merck & Co Inc, Rahway, NJ, USA). The resulting solution was transferred to a Teflon‐lined stainless‐steel autoclave and maintained at 120 °C for 12 h without stirring. Afterward, the precipitate was collected and rinsed several times with deionized water and ethanol using ultrasonication. Finally, the sample was freeze‐dried overnight.

### Conventional Electrochemical Measurements

EC measurements were performed by a conventional three‐electrodes setup. A graphite rod served as the counter electrode and a Hg/HgO electrode immersed in a 4.24 m KOH solution was used as reference (PINE research, Durham, NC 27 705, USA). All potentials reported in this work are converted to the reversible hydrogen electrode (RHE) using the formula E(V vs RHE) = E(V vs MOE) + E(MOE vs Standard hydrogen electrode (SHE)) + 0.059*pH, where E(MOE vs SHE) = 0.107 V was obtained upon calibration toward a SCE master electrode in 0.1 m KOH, yielding the scaling relationship E(V vs RHE) = E(V vs MOE) + 0.874. Similar results (0.870 V) were obtained using the crossover point between hydrogen oxidation and reduction in H_2_‐saturated 0.1 m KOH with Pt electrodes. The electrocatalytic properties were tested using an ink prepared by mixing 5 mg of active material with 2 mg of conductive carbon (Vulcan) and 25 µL of a 5% Nafion solution in a 1 mL Milli‐Q water/ethanol solution (1:1). An amount of 50 µL of ink was drop‐casted on 3 mm glassy carbon electrodes. A 0.1 m KOH solution was used as the electrolyte for all experiments.

### TEM

TEM and high‐angle annular dark‐field scanning transmission electron microscopy (HAADF‐STEM) were used to characterize the morphology and microstructure of the samples. These analyses were carried out using a JEOL F200 TEM. Elemental analysis and mapping were performed with a JEOL 100 mm^2^ silicon drift EDX spectrometer. Carbon‐supported gold grids, 400 mesh size, were used for the preparation of the sample and ex‐situ electrochemistry.

### NEXAFS and *Operando* NEXAFS

NEXAFS spectra at Co L_2,3_‐, O K‐, and Al K‐ edges were measured both in ex‐situ UHV and in *operando* conditions at the BACH beamline at the Elettra synchrotron facility in Trieste, Italy, recorded using synchrotron radiation linearly polarized in the horizontal plane, with an energy resolution better than 250 meV. NEXAFS acquired ex‐situ were measured in FY mode by collecting the signal with a multichannel plate (MCP) detector (Hamamatsu, F4655‐13), while *operando* NEXAFS were measured in FY with a photodiode (IRD, AXUV100G). *Operando* experiments were performed using an upgraded version of the EC‐cell,^[^ ^60^ ^]^ which is currently installed on a BACH branch‐line entirely dedicated to in‐situ and *operando* electrochemical experiments. The EC‐cell is equipped with a three‐electrode system and a soft X‐ray transparent membrane window (gold‐coated Si_3_N_4_ membrane, 100 nm thick) to isolate the vacuum environment from the wet sample conditions. See a more thorough discussion of the setup in supporting information and Figure S1 (Supporting Information). The cell includes an Ag/AgCl reference electrode, a platinum wire counter electrode, and the cell window as a working electrode, where a CoAl‐LDH ink, prepared as described above for conventional EC, was deposited as a thin film. Microvalves located at the base of the EC‐cell enable the electrolytic solution to be refreshed during the experiments using a peristaltic pump.

This setup enables the measurement of NEXAFS spectra in working conditions at selected applied potentials and in the presence of an electrolyte. Photon energies were calibrated by XPS, measuring the kinetic energies of the Au 4*f* core level on a clean gold foil.

### *Operando* AFM

*Operando* AFM measurements were conducted using a commercial Keysight 5500 AFM instrument. Since the experiments were carried out in a KOH solution, a specialized nosecone with a plastic viewport was used in place of the standard optical glass. This adjustment was necessary to prevent the optical glass from being etched or matted by prolonged immersion in the KOH electrolyte. The laser beam used for cantilever deflection passed through this plastic viewport, ensuring optimal beam intensity for the duration of the experiment.

All the reported images were collected in tapping mode to ensure high‐resolution data without physically contacting the sample surface. For these measurements, gold‐coated AFM tips provided by TipsNano (NSG30, ω_air_ = 300 kHz, k = 70 N m^−1^) were utilized. These tips offer enhanced performance and durability in liquid environments. The mean resonance frequency of the cantilever in the liquid environment was ≈130 kHz, which is suitable for tapping mode imaging and provides high sensitivity and precision in the measurements. This careful consideration of materials and measurement conditions is crucial for obtaining accurate and reliable AFM data, particularly in corrosive environments like KOH.

The Keysight 5500 AFM is equipped with an integrated potentiostat, enabling the performance of combined electrochemical and AFM measurements. The quality and effectiveness of this setup are detailed in ref. [61].

### Computational Details

All numerical experiments have been performed by employing the ORCA program package^[^ ^62^ ^]^ on 2 models. In the first model, six water molecules are added to the Co atom to reproduce the octahedral coordination, while in the second model, four water molecules are added for the tetrahedral coordination. Both these models are optimized for Co^2+^ and Co^3+^ oxidation states and high spin and low spin configurations. The high spin species resulted to be the most stable. Molecular properties have been evaluated using the hybrid Becke3–Lee–Yang–Parr (B3LYP)^[^ ^63^, ^64^ ^]^ (i.e., by combining a standard generalized gradient (VWNIII)^[^ ^65^ ^]^ with a part (20%) of the Hartree–Fock exchange and by adopting the def2‐TZVP(‐f) basis set.^[^ ^66^, ^67^ ^]^ Co L_2,3_ spectra have been evaluated using the DFT/ROCIS method,^[^ ^19^, ^66^, ^67^ ^]^ which includes spin‐orbit coupling (SOC) in a molecular Russell–Saunders fashion. Due to the strong 2p SOC in the final state manifold, excited states with spin quantum numbers different from the ground state had to be computed. The combined use of DFT and CI needs a set of three semi‐empirical parameters (c_1_, c_2_, c_3_). As a result, we adopted the following values for them that include c_1_ = 0.18, c_2_ = 0.20, and c_3_ = 0.40. This set of parameters is previously used by one of us to simulate Co L_2,3_ spectra.^[^ ^21^ ^]^ Throughout the calculations, the resolution of the identity approximation^[^ ^68^, ^69^, ^70^ ^]^ has been used with the def2‐TZVP/J basis set.^[^ ^66^ ^]^ Moreover, the zeroth order regular approximation (ZORA) has been adopted to treat scalar relativistic effects.^[^ ^71^ ^]^ Numerical integrations for all the calculations have been carried out on a dense Lebedev grid (302 points).^[^ ^72^ ^]^ Simulated spectra of both oxidation states and coordination have been shifted by the same amount (13.6 eV) to superimpose the highest intensity feature of the experimental and simulated Co^3+^ L_3_‐edge octahedrally coordinated, which does not suffer from extra broadening and distortion due to the Coster–Kronig Auger decay process.^[^ ^73^ ^]^ A Gaussian broadening factor of 1.0 eV has been applied throughout to model the Co L_2,3_‐edge NEXAFS spectra. These simplified models were previously used^[^ ^23^ ^]^ to simulate transition metal L_2,3_ spectra. Despite the large simplicity of the models, they include the most relevant aspects to simulate the metal L_2,3_ spectra properly: i) the Co is bonded to oxygen atoms as in the real systems and ii) the coordinative environment (octahedral of tetrahedral) is considered.

## Conflict of Interest

The authors declare no conflict of interest.

## Supporting information



Supporting Information

## Data Availability

The data that support the findings of this study are available from the corresponding author upon reasonable request.
