# Supplementary material for: Operando Exploration of CoAl‐LDH: Transformations Driving Alkaline Oxygen Evolution Reaction
Source: Small. 2025 Apr 2;21(19):2412351. doi: 10.1002/smll.202412351 (PMC12067176; doi:10.1002/smll.202412351)
Supplement: Supplementary file 1 — Supporting Information [file SMLL-21-2412351-s001.pdf]

NANO · MICRO
small

Supporting Information

for *Small*, DOI 10.1002/smll.202412351

Operando Exploration of CoAl-LDH: Transformations Driving Alkaline Oxygen Evolution Reaction

Mattia Cattelan, Jijin Yang, Leonardo Cielo, Silvia Nappini, Silvia Carlotto, Marco Nalesso, Ilargi Napal Azcona, Rossella Yivlialin, Xiaoming Sun, Gianlorenzo Bussetti, Elena Magnano and Stefano Agnoli**

Supporting Information

Operando Exploration of CoAl-LDH: Transformations Driving Alkaline Oxygen Evolution Reaction

Mattia Cattelan[a,b,c,*], Jijin Yang[a], Leonardo Cielo[a], Silvia Nappini[d], Silvia Carlotto[a,b,c], Marco Nalesso[a], Ilargi Napal Azcona[d,e], Rossella Yivlialin[f], Xiaoming Sun[g], Gianlorenzo Bussetti[f], Elena Magnano[d,h] and Stefano Agnoli[a,b,c,**]

[a] Department of Chemical Sciences, University of Padova, via Marzolo 1, Padova, 35131, Italy

[b] INSTM Istituto Nazionale Scienza e Tecnologia dei Materiali, Padova Research Unit, 50121, Firenze, Italy

[c] CIRCC Consorzio Interuniversitario Reattività e Catalisi, Padova Research Unit, 70126, Bari, Italy

[d] CNR - Istituto Officina dei Materiali (IOM), Trieste, S.S. 14 km 163.5, Trieste, 34149, Italy

[e] Physics Department, P.le Europa 1, 34127 Trieste, University of Trieste, Italy

[f] Department of Physics, Politecnico di Milano, p.za Leonardo da Vinci 32, I-20133 Milan, Italy

[g] State Key Laboratory of Chemical Resource Engineering, Beijing Advanced Innovation Center for Soft Matter Science Engineering, College of Chemistry, Beijing University of Chemical Technology, Beijing, 100029, People's Republic of China

[h] Nanotechnology Research Laboratory, Faculty of Engineering, University of Sydney, Camperdown 2006, Australia

* Corresponding author: e-mail address mattia.cattelan@unipd.it

** Corresponding author: e-mail address stefano.agnoli@unipd.it

Operando NEXAFS setup

Operando experiments were performed using an upgraded version of the EC-cell[1] which is currently installed on a branch-line of the BACH beamline entirely dedicated to in-*operando* electrochemical experiments (manuscript in preparation). The liquid electrolyte is confined within the EC-cell, which utilizes a three-electrode system and a soft X-ray transparent window (gold-coated Si_3N_4 membrane, 100 nm thick, window size 1 mm x 1 mm) to isolate the vacuum environment from the wet sample conditions. The gold coating, consisting of a 5 nm Ti layer (to improve Au adhesion) and a 15 nm Au layer, is crucial for ensuring good electrical contact at the cell window, which serves as the working electrode (WE), where a thin film of CoAl-LDH ink, prepared as previously described for conventional EC, is deposited. The cell body is made entirely of PEEK, which is chemically resistant to commonly used electrolytes. The setup includes a miniaturized leak-less Ag/AgCl reference electrode (RE), a platinum plate counter electrode (CE), and the cell window, coated with the catalyst, as the working electrode (WE) (see Fig. S1), all connected to an Emstat3+ (PalmSens) potentiostat.

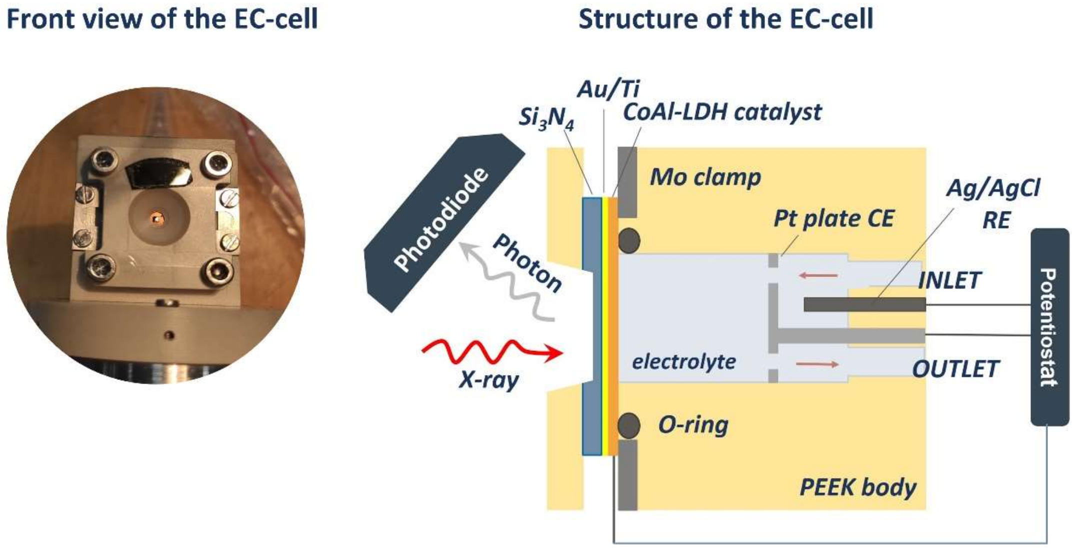

Figure S1. A front-view image and a schematic drawing of the microfluidic EC-cell for *operando* s-XAS experiments. The cell is equipped with a 3-electrodes system: Au-coated Si_3N_4 (WE), Pt plate (CE), Ag/AgCl electrode (RE). The cell body, made of PEEK, is sealed with an O-ring (FFKM, NBR, or EPDM, depending on the electrolyte and pH).

This design supports various electrochemical experiments, such as cyclic voltammetry (CV), linear sweep voltammetry (LSV), electrodepositions, and electrochemical impedance spectroscopy (EIS). Two interlocked microvalves at the base of the EC-cell allow the electrolytic solution to be refreshed during experiments using a peristaltic pump, while also controlling the liquid volume in the cell during measurements. This setup enables the acquisition of s-XAS spectra under working conditions at selected applied potentials and in the presence of an electrolyte. Photon energies were calibrated using XPS by measuring the kinetic energies of the Au 4f core level on a clean gold foil.

OER Tests in Conventional EC three-electrodes Setup

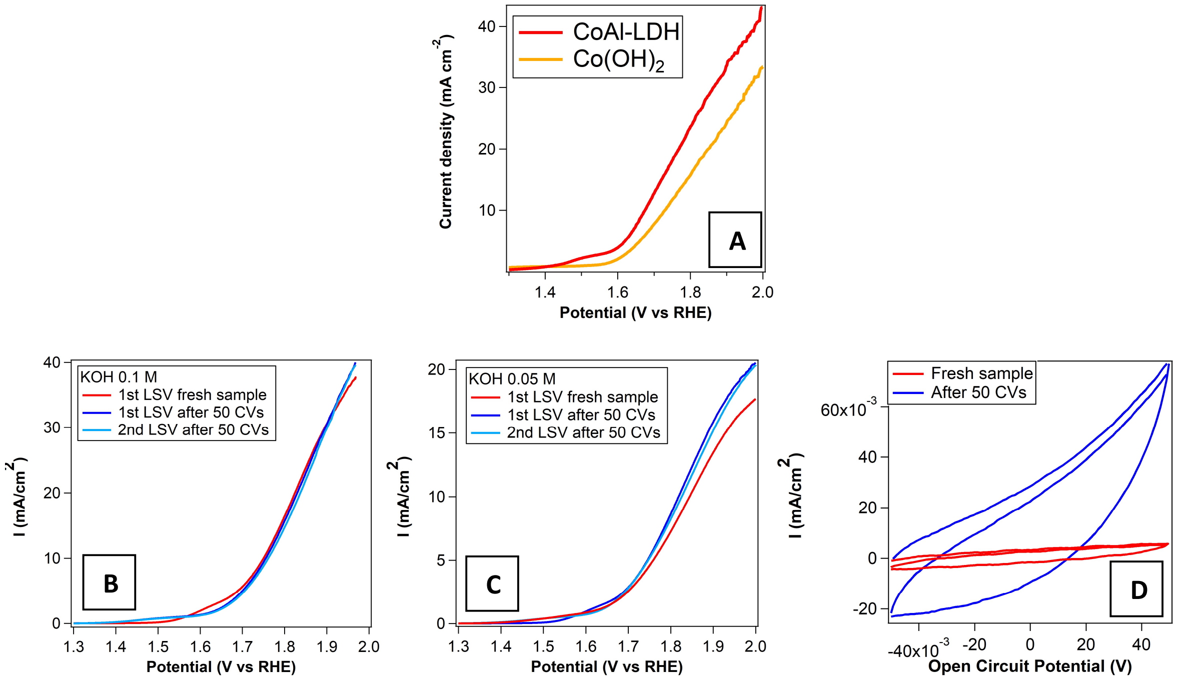

Fig. S2. (A) LSV at 20 mV/s in 0.1 M KOH to test the OER performance of CoAl-LDH and similarly synthesized $Co(OH)_2$. (B) LSV at 20 mV/s in 0.1 M KOH before (red) and after 50 CVs (first scan in blue, second scan in cyan) performed from 0.7 to 1.7 V at 50 mV/s. (C) Same as B but conducted in 0.05 M KOH. (D) CV around OCP (±0.05 V) of fresh CoAl-LDH (red) and after 50 CVs as described in B (blue).

NEXAFS spectra of as-prepared sample and ex-situ post EC accelerated aging

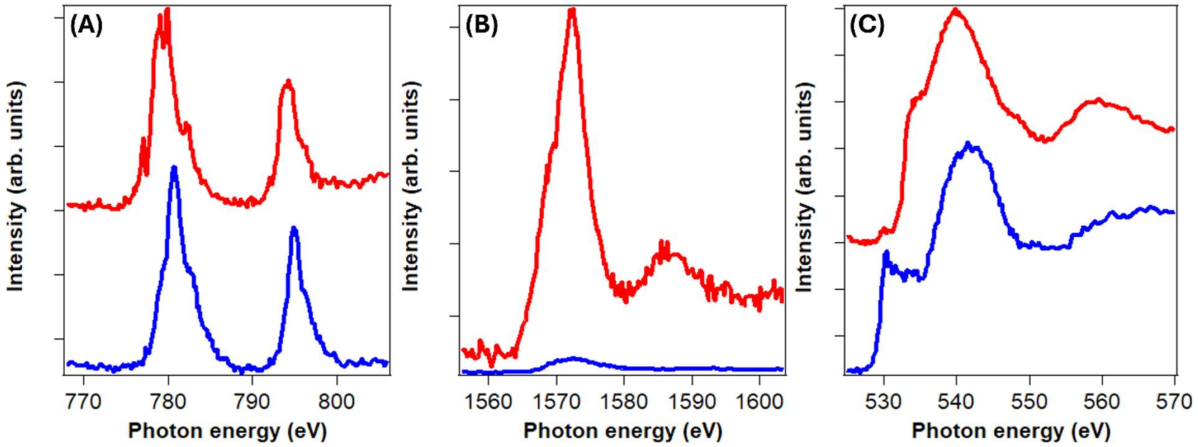

Fig. S3. Pre(red)-post(blue) OER aging conventional NEXFAS fluorescence spectra, (A) Co $L_{2,3}$-edge, (B) Al K-edge (C) O K-edge

NEXAFS references and comparisons

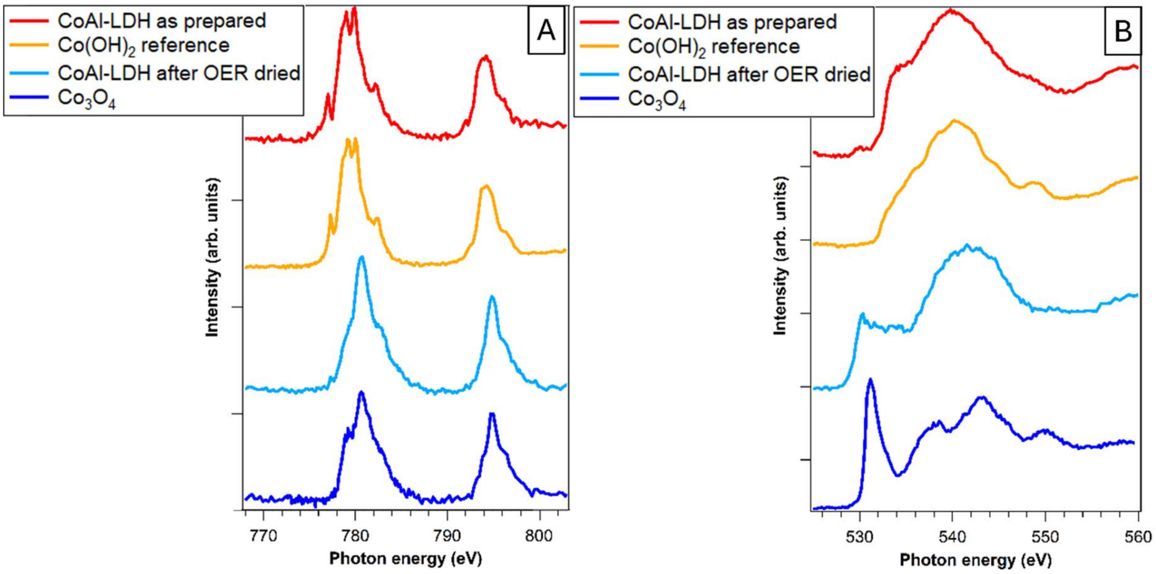

Fig. S4. Conventional NEXAFS for (A) Co $L_{2,3}$- and (B) O K-edge in FY, using an MCP detector (for CoAl-LDH (red), β-$Co(OH)_2$ (yellow), CoAl-LDH after OER dried (light blue), Co_3O_4 (dark blue).[2]

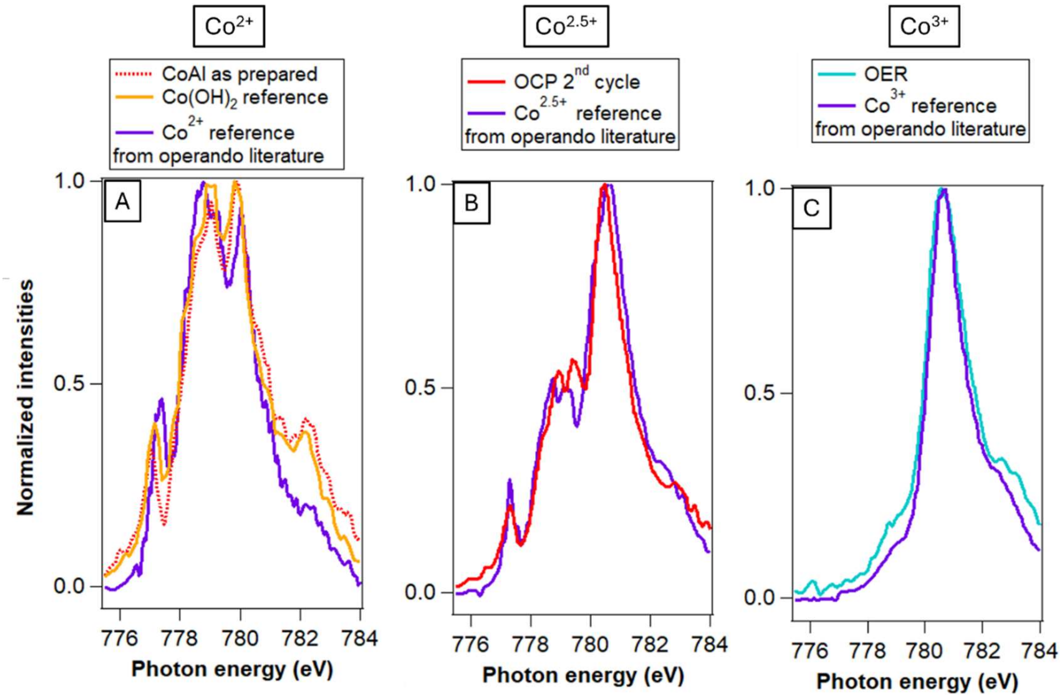

Fig. S5. Comparison of NEXAFS Co L_3 edge: (A) CoAl-LDH spectrum (red dashed line), $Co(OH)_2$ reference[2] (yellow line), and *operando* Co^{2+} adapted from Ref.[3] (purple line). (B) This work CoAl-LDH at OCP after 2nd CV cycle (red line), and $Co^{2.5+}$ adapted from Ref.[3] (purple line). (C) This work CoAl-LDH at OER (light blue line), and Co^{3+} *operando* adapted from Ref.[3] (purple line). Purple spectra adapted from Ref. [3].

Simulation of Co^{2+} octahedrally coordinated by oxygen

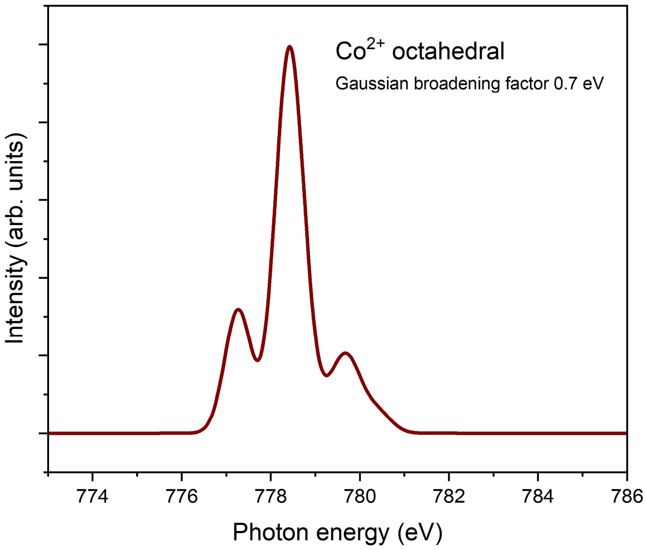

Fig. S6. Simulated Co^{2+} octahedrally coordinated by oxygen with Gaussian brodening of 0.7 eV.

Average oxidation state calculation example

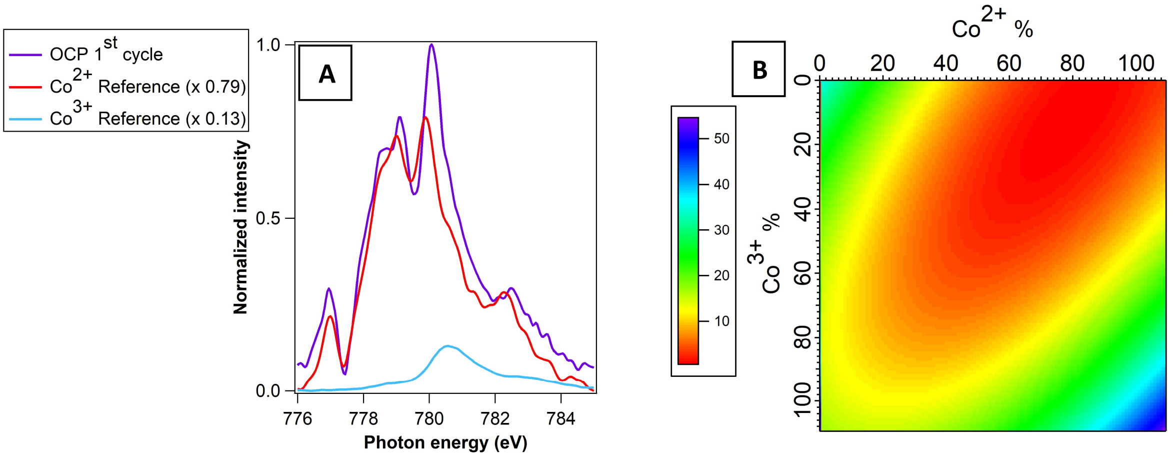

Fig. S7.(A) *Operando* Co L_3-edge spectrum measured during the first OCP cycle for CoAl-LDH (purple). The Co^{2+} and Co^{3+} reference spectra are shown in red and light blue, respectively (see main text for details on the reference options). The reference spectra are scaled to achieve the best final combination. (B) Square difference of the linear combination of the Co^{2+} and Co^{3+} reference spectra. The optimal combination corresponds to the lowest value, indicated in red in the figure. For this specific sample, the best fit yielded a composition of 79 % of Co^{2+} and 13 % of Co^{3+}, resulting in an average oxidation state of approximately 2.1, as calculated using the formula described in the main text.

Simulation for Co_3O_4

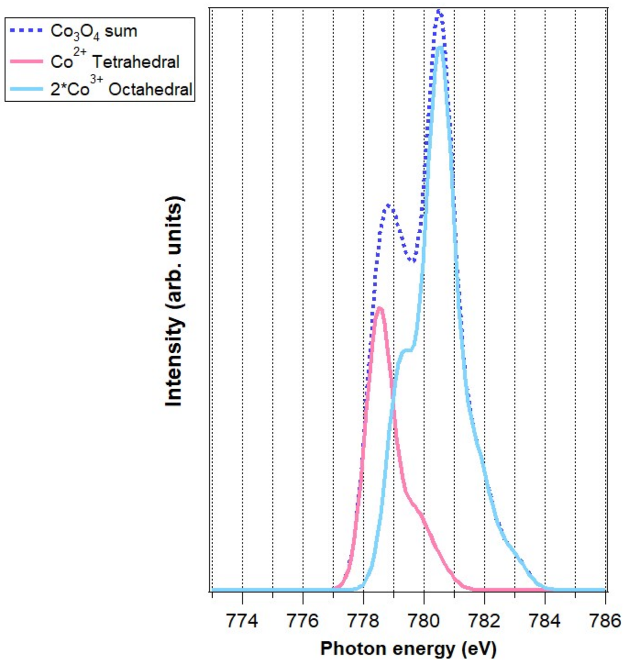

Fig. S8. Simulated spinel Co_3O_4 phase obtained by summing one Co^{2+} tetrahedral (pink) and two Co^{3+} octahedral (light blue) components.

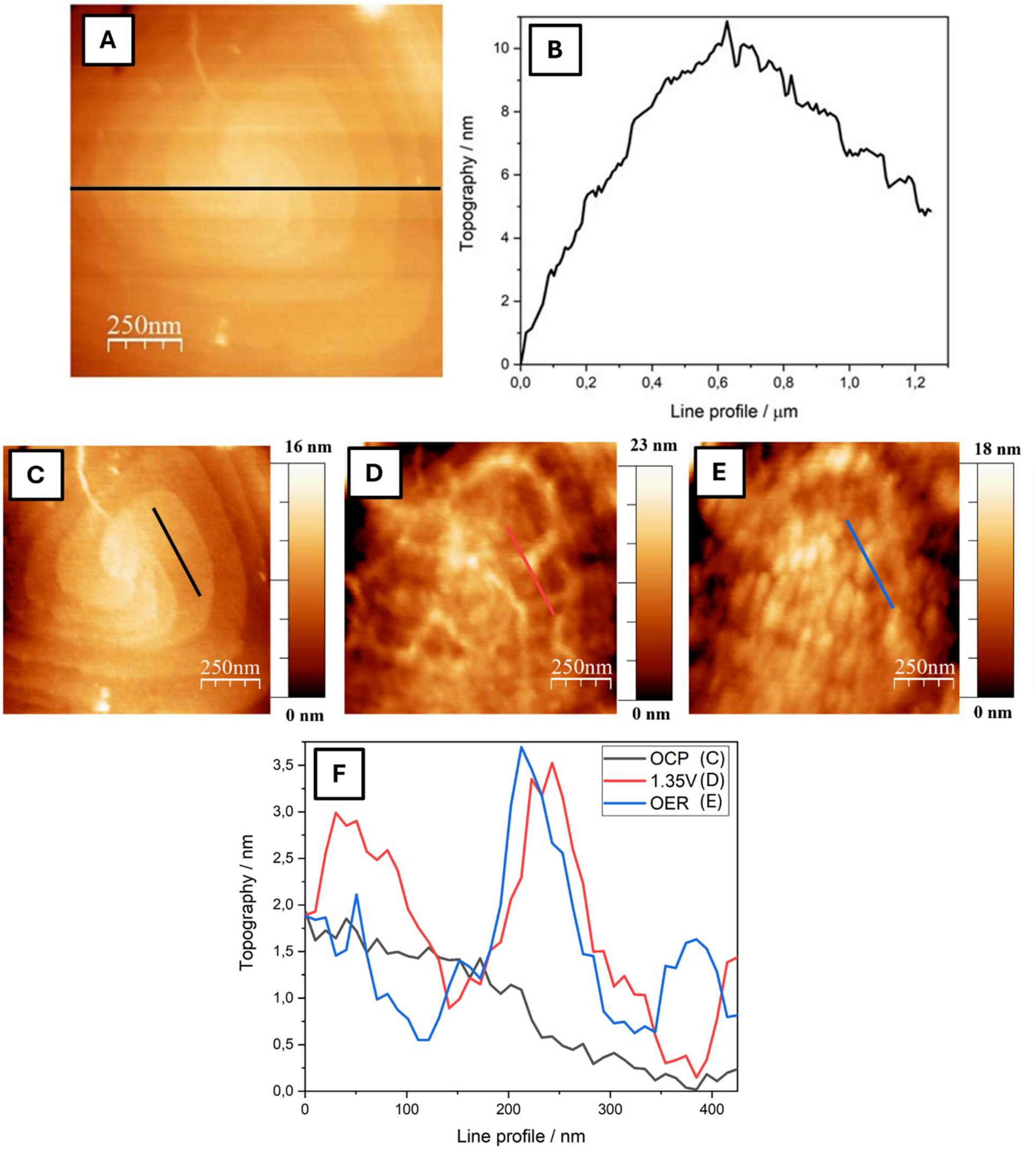

Fig. S9. A) Topographic image at the OCP at the center of the hexagonal flake. The black line represents the path for the profile reported in B. B) Topographic line profile across the screw dislocation, clearly showing dislocation steps with an approximate height of 1 nm. Comparison of the central part of the hexagon at OCP (C), 1.35 V (D), and OER (E). The topographic profiles (F) were traced along the lines depicted in C, D and E. The line position in C was chosen to be on a flat area of the screw dislocation, reporting the same line profile in D and E allow to observe the drastic changes as a function of the EC potential.

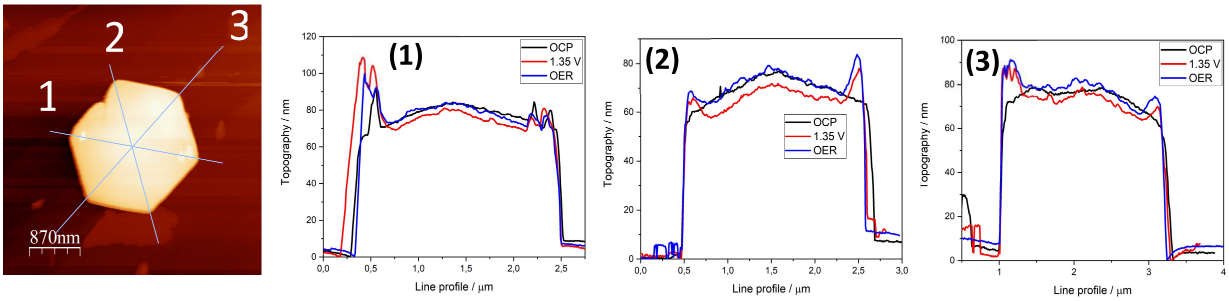

Fig. S10. AFM topographic images and line profile at the OCP, 1.35 V and during OER. It is clearly visible, especially in line profile (3), that the basal plane roughness increases in OER conditions. The edge rise is observable in all the line profiles and starts at the intermediate potential of 1.35 V.

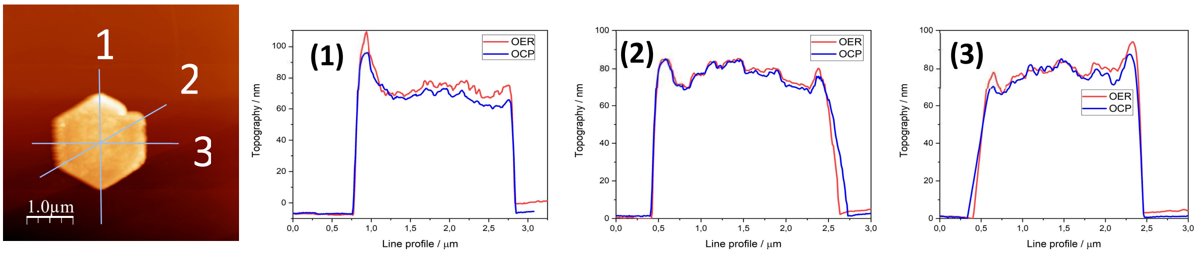

Fig. S11. AFM topographic images and line profile from OER to OCP. Traced line profiles underline the good morphological stability, i.e. corrugation and edge rising remains stables even at OCP conditions.

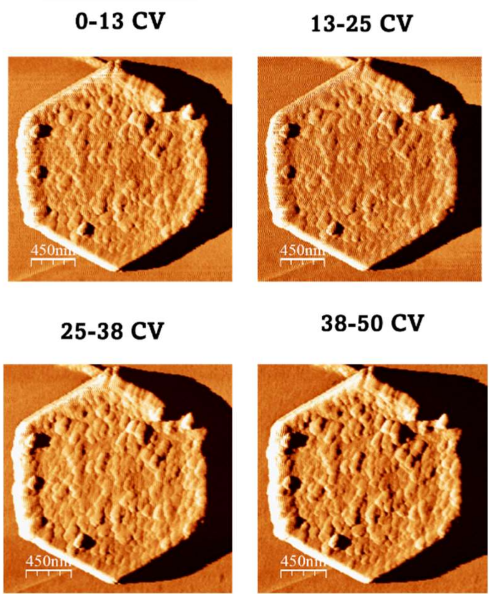

Fig. S12. AFM phase image during 50 CV cycles from OCP to OER conditions after extensive OER exposure, see main text. Scan speed was set at 50 mV/s, which means that during every scan the system performs 12.5 CV. CV numbers that occurred during the scan are listed on top of the images. From this analysis, the system resulted to be stable, and the dimension of the particles does not significantly change.

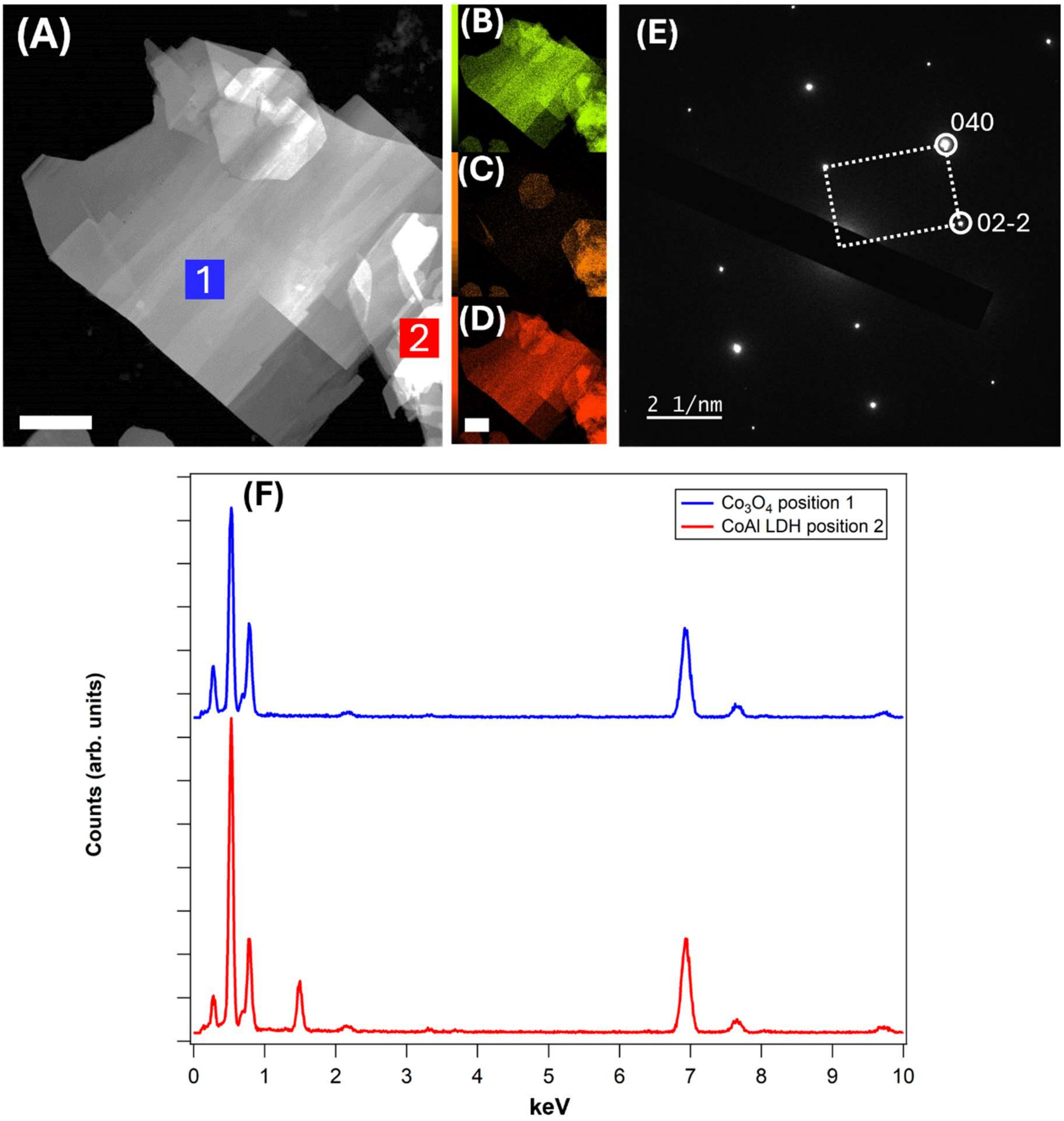

Fig. S13. TEM panel *ex-situ*. (A) Secondary electron image of CoAl-LDH after accelerated OER aging, (B) Co K edge map, (C) Al K edge map, (D) O K edge map, all scale bars: 1 µm (E) SAED with indexed spots. (F) EDX spectra of point 1 and 2 in (A).

XRD and XPS

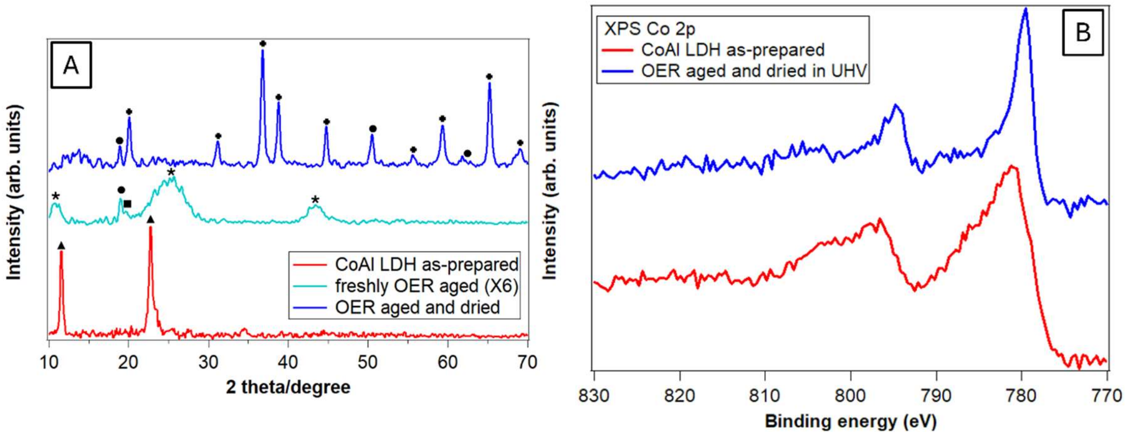

Fig. S14. (A) XRD spectra for CoAl-LDH in its as-prepared state, freshly EC-aged sample, and extensively dried sample are shown in red, cyan, and blue, respectively. Reflections corresponding to CoAl-LDH[4], β-$Co(OH)_2$, β-CoOOH, and Co_3O_4[5] are indicated by triangles, circles, squares, and crosses, respectively. Asterisks denote the broad reflections from Vulcan carbon. (B) XPS spectra of CoAl-LDH and the EC-aged and dried sample are shown in red and blue, respectively.

Regarding the photoemission data in Fig. S14B, the red spectrum clearly indicates the presence of Co^{2+}, as evidenced by the shake-up feature around 785 eV,[6] consistent with $Co(OH)_2$. The binding energy at 781.2 eV is slightly higher than that of the reference for $Co(OH)_2$,[6] likely due to the positive charge introduced into the LDH structure by the presence of Al. We cannot exclude some charging effects due to the poor electron conductivity of Nafion in the ink composition. Indeed, the shake-up feature and the main line of Co^{2+} are usually well separated. These charging issues were not observed in the NEXAFS spectra, which is why we used this technique for operando measurements.

After EC aging, the spectrum aligns with Co_3O_4.[6] However, it is important to note that distinguishing between the spinel phase and CoOOH using this technique is challenging,[7] and it is worth noting that a thin layer of CoOOH can form on Co_3O_4 particles after OER, as recently reported. [8] This makes it even more difficult to understand the bulk transformation of the material via XPS, which is intrinsically more surface-sensitive than NEXAFS.

References

[1] S. Nappini, L. D'Amario, M. Favaro, S. Dal Zilio, F. Salvador, E. Betz-Güttner, A. Fondacaro, I. Píš, L. Romanzin, A. Gambitta, F. Bondino, M. Lazzarino, E. Magnano, *Review of Scientific Instruments* **2021**, *92*, 015115.

[2] T. Tran-Phu, R. Daiyan, J. Leverett, Z. Fusco, A. Tadich, I. Di Bernardo, A. Kiy, T. N. Truong, Q. Zhang, H. Chen, P. Kluth, R. Amal, A. Tricoli, *Chemical Engineering Journal* **2022**, *429*, 132180.

[3] J. T. Mefford, A. R. Akbashev, M. Kang, C. L. Bentley, W. E. Gent, H. D. Deng, D. H. Alsem, Y.-S. Yu, N. J. Salmon, D. A. Shapiro, P. R. Unwin, W. C. Chueh, *Nature* **2021**, *593*, 67.

[4] Y.-J. Wang, J.-Y. Zhang, S.-S. Hou, J.-X. Wu, C. Wang, Y.-M. Li, G.-Y. Jiang, G.-Q. Cui, *Pet Sci* **2022**, *19*, 3080.

[5] Y.-C. Liu, J. A. Koza, J. A. Switzer, *Electrochim Acta* **2014**, *140*, 359.

[6] M. C. Biesinger, B. P. Payne, A. P. Grosvenor, L. W. M. Lau, A. R. Gerson, R. St. C. Smart, *Appl Surf Sci* **2011**, *257*, 2717.

[7] T. Kosmala, L. Calvillo, S. Agnoli, G. Granozzi, *ACS Catal* **2018**, *8*, 2343.

[8] C. Qiu, F. Maroun, M. Bouvier, I. Pacheco, P. Allongue, T. Wiegmann, C. H. Scharf, V. de Manuel-Gonzalez, F. Reikowski, J. Stettner, O. M. Magnussen, *ChemCatChem* **2024**, e202400988.
